# The effector candidate repertoire of the arbuscular mycorrhizal fungus Rhizophagus clarus

**DOI:** 10.1186/s12864-016-2422-y

**Published:** 2016-02-09

**Authors:** Kinga Sędzielewska Toro, Andreas Brachmann

**Affiliations:** Genetics, Faculty of Biology, Ludwig-Maximilians-University Munich, Großhaderner Straße 2-4, 82152 Planegg-Martinsried, Germany

**Keywords:** AMF, Effector, *in silico* pipeline, Plant, *Rhizophagus*, Symbiosis

## Abstract

**Background:**

Arbuscular mycorrhizal fungi (AMF) form an ecologically important symbiosis with more than two thirds of studied land plants. Recent studies of plant-pathogen interactions showed that effector proteins play a key role in host colonization by controlling the plant immune system. We hypothesise that also for symbiotic-plant interactions the secreted effectome of the fungus is a major component of communication and the conservation level of effector proteins between AMF species may be indicative whether they play a fundamental role.

**Results:**

In this study, we used a bioinformatics pipeline to predict and compare the effector candidate repertoire of the two AMF species, *Rhizophagus irregularis* and *Rhizophagus clarus*. Our *in silico* pipeline revealed a list of 220 *R. irregularis* candidate effector genes that create a valuable information source to elucidate the mechanism of plant infection and colonization by fungi during AMF symbiotic interaction. While most of the candidate effectors show no homologies to known domains or proteins, the candidates with homologies point to potential roles in signal transduction, cell wall modification or transcription regulation. A remarkable aspect of our work is presence of a large portion of the effector proteins involved in symbiosis, which are not unique to each fungi or plant species, but shared along the Glomeromycota phylum. For 95 % of *R. irregularis* candidates we found homologs in a *R. clarus* genome draft generated by Illumina high-throughput sequencing. Interestingly, 9 % of the predicted effectors are at least as conserved between the two *Rhizophagus* species as proteins with housekeeping functions (similarity > 90 %). Therefore, we state that this group of highly conserved effector proteins between AMF species may play a fundamental role during fungus-plant interaction.

**Conclusions:**

We hypothesise that in symbiotic interactions the secreted effectome of the fungus might be an important component of communication. Identification and functional characterization of the primary AMF effectors that regulate symbiotic development will help in understanding the mechanisms of fungus-plant interaction.

**Electronic supplementary material:**

The online version of this article (doi:10.1186/s12864-016-2422-y) contains supplementary material, which is available to authorized users.

## Background

Arbuscular mycorrhizal fungi (AMF) live in an obligate symbiosis with the roots of around two-thirds of all studied land plant species, i.e. Angiosperms, Gymnosperms, Pteridophytes and some Bryophytes [1], and in one known case with cyanobacteria [2]. This interaction has a major impact on the entire soil ecosystem and plays a crucial role in agricultural systems by increasing plant tolerance to biotic and abiotic stresses [3]. In the field, AMF form mycelial networks providing extensive connections between roots of different plant species and mediating interactions among plants. These networks significantly improve the rhizosphere soil stability [4]. The major benefit for plants upon mycorrhization is a more efficient nutrient uptake from the soil [5–8]. In exchange, AMF obtain photoassimilates from their hosts [9].

All AMF belong to the phylum Glomeromycota which has been divided into four orders: the *Glomerales*, the *Diversisporales*, and the two ‘ancestral’ lineages, *Paraglomerales* and *Archaeosporales* [10]. They may have started to diverge over 600 Mya. The earliest spore fossils have been found together with the first land plants (455–460 Mya) and resemble present AMF morphological structures [11]. It has been suggested that AMF played a crucial role for the adaptation of phototrophs to the terrestrial environment [1]. The AM symbiosis persisted morphologically unchanged throughout the complete evolutionary development within the plant phylum from haploid gametophytes to diploid sporophytes [12]. Additionally, one single AMF species can often be used to inoculate dicotyledons, monocotyledons and ferns, and one plant species can be mycorrhized by several AMF species. Therefore, AMF are considered not to be host specific and there is no evidence for evolution of host specificity [12]. This finding is extremely remarkable considering the obligate biotrophic life style of AMF. Thus, the mechanism of plant infection and colonization seems to be ancient and conserved within AMF.

Especially during infection, pathogenic and symbiotic plant-microbe interactions show striking similarities, suggesting commonalities in the underlying regulation. For example, pathogenic and AM fungi develop analogous feeding structures, haustoria and arbuscules, respectively [13]. Transcriptome profiles of plant cells hosting these structures indicate activated auxin signalling and increased plant metabolism in response to both, pathogenic and symbiotic fungi [14]. The transcriptome sequencing project of the model AM fungus, *Rhizophagus irregularis* DAOM197198, showed that only a limited set of cell wall degrading enzymes is expressed during invasive growth, presumably aimed to avoid a major release of polysaccharide fragments, thereby, their detection by the plant immune system [15]. This is analogous to the gene expression patterns in obligate biotrophic pathogens such as the fungus *Blumeria graminis* and the oomycete *Albugo laibachii* [15]. Recent studies of plant-mutualistic ectomycorrhizal fungi interactions point in the same direction: genes encoding effector proteins were shown to play a key role in host colonization by controlling the plant immune system [16].

An important factor in plant-microbe interactions are microbial effector proteins released to alter plant cell structure or function, allowing successful infection by suppressing the host defence response [17]. In plant pathogenic fungi and oomycetes two classes of effectors are known. The first class comprises apoplastic effectors that are secreted into the extracellular space. They exert their function by interfering with the plant defence in the apoplast and contain small cysteine-rich proteins (SCRs) and inhibitors of plant extracellular hydrolases including proteases (e.g. *Cladosporium fulvum* avr2, avr9) [18, 19], glucanases and plant cell wall degrading enzymes [17]. The second class encompasses intracellular effectors that are translocated into the host cell and target different subcellular compartments. These effectors may be recognized inside the plant cell by intracellular NB-LRR (nucleotide-binding site leucine-rich repeat) proteins encoded by *R* resistance genes, which results in the induction of effector-triggered immunity (ETI) [20].

Most of the identified eukaryotic pathogenic effectors do not contain domains or homologies to proteins with known function; therefore their roles remain unclear. Although in oomycete pathogens the RXLR motif has been implicated in translocation of effectors into the host cell, to date no universal host-transportation motif has been identified for fungi [21, 22]. Fungal and oomycete effector genes seem to be at the forefront of the arms race between host and pathogen: their high rate of nonsynonymous sequence substitutions is a strong signature of positive selection. This leads to a high amino acid polymorphism, so that, from the 536 predicted RXLR genes in the oomycete *Phytophthora infestans*, only 16 belong to the “core orthologs” present in other *Phytophthora* species [22].

In contrast to pathogenic systems, there is a vast gap of knowledge on effector proteins in symbiotic fungal-plant interactions [23]. There are indications that also here they may play a role in modulating the plant immune response and consequently the efficiency of fungal infection and colonization of plant cells [16, 24]. Kloppholz and colleagues [24] discovered in the AM fungus *R. irregularis* the secreted effector protein, SP7, influencing fungal development within plant roots. SP7 interacts with the pathogenesis-related transcription factor ERF19 in the *Medicago truncatula* nucleus, and its constitutive expression in roots led to increased mycorrhization and reduced expression of plant defence genes.

Since infection and colonization processes appear to be conserved within AMF, we would also expect effectors playing a role during AM symbiosis to be conserved among AMF species. The level of conservation could then be indicative of whether they play a major role or have instead a rather supplemental function during the symbiosis establishment. Therefore, this scenario would be the exact opposite situation as in pathogenic plant-microbe interactions.

In this study, we predicted and compared the effector candidate repertoire of the two AMF, *R. irregularis* and *Rhizophagus clarus*. To this end, we have used the published *R. irregularis* genome [25] and a *R. clarus* genome draft generated by Illumina high-throughput sequencing. For most of the predicted effector candidates, we were able to identify homologs in both *Rhizophagus* species. In addition, for several of them we could postulate potential roles in symbiosis by means of gene ontology.

## Methods

### *R. clarus* monoxenic culture

*R. clarus* in vitro culture was established as described by Declerck et al. [2]. Chicory Ri-T DNA transformed root organ culture (*Cichorium intybus*, Munich, Germany) was cultivated with a single-spore isolate of *R. clarus* MUCL46238 on one side of two-compartment Petri dishes containing MRS medium with 0.5 % phytagel (Sigma-Aldrich). Fungal hyphae, but not chicory roots, were allowed to grow over the plastic barrier to the second compartment. Plates were incubated in the dark at 26 °C for up to six months until the development of secondary spores.

Extraradical mycelia and secondary spores were recovered from the root-free plate compartments by dissolving the medium in 10 mM citrate buffer [2]. The fungal material was collected under sterile conditions [26] and stored at −20 °C or −80 °C until used.

### *R. clarus* genomic DNA sequencing

Genomic DNA was extracted from 100 mg spores and hyphae using the DNeasy Plant Mini Kit (Qiagen) following manufacturer’s instructions. The genomic sequencing library was constructed with the Nextera DNA Sample Preparation Kit (Illumina), which allows for low gDNA input (50 ng) and does not need prior DNA shearing. The library was quality controlled by analysis on an Agilent 2000 Bioanalyzer with Agilent High Sensitivity DNA Kit (Agilent Technologies) for fragment sizes of around 200–400 bp. Sequencing on a MiSeq sequencer (Illumina) (2x150 bp paired-end sequencing) was performed in the Genomics Service Unit (LMU Biocenter, Martinsried, Germany), yielding about 72 Mio reads and 8 Gb of primary sequence*.* An initial draft with approx. 53-fold coverage of the *R. clarus* genome was assembled using CLC Genomics Workbench v6.0 (Qiagen) with the following parameters: word size, 24; bubble size, 50; mismatch cost, 2; insertion cost, 3; deletion cost, 3; length fraction, 0.8; similarity fraction, 0.8; mapping mode, map reads back to contigs; minimum contig length, 1,000; mismatch cost, 2. Contaminating sequences were identified by analysis with the metagenomics server, MG-RAST [27]. 80 % of the contigs passed quality control (QC) and from these, around 25 % were recognized as bacterial contamination based on sequences available in databases. Contaminating sequences were removed by mapping against genomes of *Bacillus cereus* (NC004722.1), *Bradyrhizobium elkanii* (GCA000472865.1), *Bradyrhizobium japonicum* (BA000040.2), *Legionella pneumophila* (NC006368.1), and *Sorangium cellulosum* (AM746676.1), which were closely related to the main bacterial contaminants. In addition, all contigs with a GC content > 50 % were eliminated. 84 % of the raw sequence information could be remapped onto the final assembly. The draft assembly statistics are summarized in Additional file 1: Table S1. Completeness of the draft genome assembly was assessed using the BUSCO software tool [28] for genomes with the fungal gene set and with default parameters (Additional file 1: Table S1).

### Bioinformatic prediction of the *R. irregularis* effectome

**Fig. 1:**
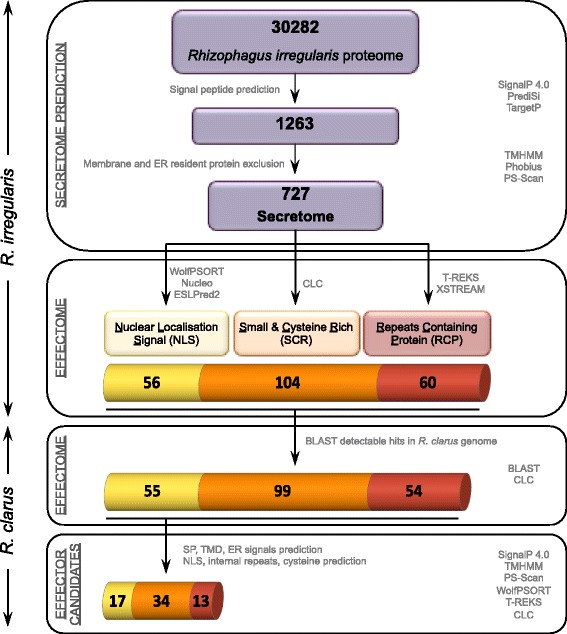
*In silico* analysis pipeline for identification of the putative effector repertoire from the genomes of two AMF, *R. irregularis* and *R. clarus*

The *R. irregularis* DAOM197198 secretome was predicted from the initial putative proteome (30,282 proteins) available at the DOE Joint Genome Institute (JGI, France) database by use of a series of domain and protein structure prediction programs. The 1,263 proteins carrying N-terminal signal peptides (SPs) were predicted using the well validated SignalP 4.0 and PrediSi programs and sub-cellular targeting using TargetP [29] with default parameters. To ensure stringent standards, only proteins predicted being secreted by at least two methods were considered further. This prediction was followed by exclusion of proteins likely to be retained into the plasma membrane in which a transmembrane domain (TMD) was forecasted by TMHMM 2.0c and Phobius [29, 30] with default parameters. Proteins in which predicted TMDs overlapped with signal peptides were not excluded. A PS-Scan analysis with the Prosite motif ‘PS00014’ (KDEL) filtered proteins that are likely to permanently reside in the lumen of endoplasmic reticulum [31]. The final number of proteins belonging to the *R. irregularis* secretome was 727 (Fig. 1).

Effector candidates were identified from the *R. irregularis* secreted proteins that fulfil at least one of the previously mentioned criteria:

(i) Proteins with nuclear localization signal (NLS). To find candidate effectors that target the plant nucleus, the *R. irregularis* secretome was searched for proteins containing a NLS using WoLF PSORT, Nucleo and ESLPred2 (Fungi) software with default parameters [32–34]. We identified 56 predicted proteins containing at least one NLS (Fig. 1).
(ii) Small and cysteine rich (SCR) proteins. Several known effectors from fungal and oomycetal plant pathogen are characterized by a length of less than 150 amino acids and a cysteine content of more than 3 % [17, 35]. In order to identify candidates achieving this criterion the cysteine content and length of *R. irregularis* secreted proteins were calculated using CLC Genomics Workbench. Within the secretome, we found a total of 104 proteins fulfilling this condition (Fig. 1).
(iii) Repeats containing proteins (RCP). Internal repeats are often found in oomycetal and fungal effectors [36]. Using the T-REKS algorithm and XSTREAM software with default parameters [37, 38] tandem repeat proteins were selected within the *R. irregularis* secretome. Sixty secreted proteins showed internal repeats (Fig. 1).

### *In silico* search for effector candidate homologs in *R. clarus*

Proteins of *R. irregularis* matching at least one effector criterium were subsequently used to predict candidate effectors in *R. clarus* (Fig. 1). Approx. 95 % (208) of *R. irregularis* effector candidates showed BLASTP (bl2seq) detectable hits with minimum similarity of 40 % within the *R. clarus* initial genome draft. Even though these proteins share sequence similarity they do not necessarily have a similar topology. Therefore, we applied the same selection criteria that were used on the *R. irregularis* secretome, filtering out proteins with TMDs and endoplasmic reticulum retention signals. Also, an additional control for the presence of SP, NLS and internal repeats was performed.

### Effector protein characterization by BLAST2GO

To characterized predicted effector proteins the BLAST2GO software was used [39]. At first, protein sequences encoded by effector candidate genes were compared with the non-redundant sequence database. Analysis were performed using the BLASTP algorithm with expect-value lower than 1.0e^−05^ recording max. 20 hits. Proteins with significant hits were classified into Gene Ontology categories by ‘Mapping’. Data from both analyses were merged into the annotation. Additionally, presence of conserved protein domains or motifs was determined using Pfam, Superfamily 1.73 software and NCBI Conserved Domain search with default parameters [40, 41].

## Results and Discussion

### Defining the *Rhizophagus* effector repertoire

To identify and classify effector candidates of the *Rhizophagus* genus we constructed a bioinformatics pipeline using current knowledge of the properties of validated fungal and oomycete plant pathogen effectors (Fig. 1). It was observed that known pathogen effector proteins from fungi and oomycetes are secreted and fulfil at least one of the following criteria: (i) they contain a nuclear localization signal (NLS), (ii) they are small and cysteine rich (SCR), (iii) they contain internal repeats (RCP), or (iv) they show similarity to haustorial (arbuscular) expressed proteins [17, 36]. For *R. irregularis* so far no inventory of arbuscular expressed proteins exist. Therefore, we restricted the effector candidate identification to the first three criteria.

Effector candidates need to carry an N-terminal signal peptide (SP) for secretion and should not be retained in the membrane or endoplasmic reticulum of the secreting organism. The *R. irregularis* DAOM197198 secretome was predicted from the initial putative proteome (30,282 proteins) available at the DOE Joint Genome Institute (JGI, USA) database by use of a series of domain and protein structure prediction programs. The predicted *R. irregularis* secretome contains 727 proteins (Fig. 1). The final number of *R. irregularis* predicted secreted proteins that fulfil at least one of the above-mentioned effector criteria was 220, representing 23 % of all *R. irregularis* putatively secreted proteins (Additional file 1: Table S2). In addition, we found 21 proteins fulfilling two but none that matched all three of the effector criteria.

Brief Gene Ontology category analyses by ProtFun 2.2 server [42] of both, secretome and effectome, showed enrichment in hormone, stress response, growth factor, and immune response related proteins (Additional file 1: Figure S1). When comparing secretome and effectome, only three differences are apparent. The 40 % increased number of hormone-related proteins observed in the effectome correlates with the finding that in AM the root architecture is modified by essential, hormone-mediated pathways that might be controlled by AMF [43]. A reduction by 45 % of proteins involved in immune response between effectome and secretome might be indicative of the symbiotic interaction between AMF and plant. The same could be true for the relative reduction of proposed receptor proteins by 60 % in the effectome. Receptors play an important role in all kind of interactions by detecting and recognising signals from pathogenic or symbiotic organisms present in the environment [44].

### The effectome is conserved

Given the central importance of secreted proteins in determining the outcome of plant-microbe encounters in other systems, it is likely that AMF effectors also have a crucial function. However, knowledge about AMF effectors and their function during symbiosis is limited and to date only one effector has been functionally characterised [24].

We wanted to address two questions: (i) How conserved is the effectome between two closely related AMF species? And (ii), does the identity of the conserved putative effectors shed light onto their potential function during the AMF colonization process? For this purpose we generated a draft genome of *R. clarus* MUCL46238 by high-throughput sequencing resulting in 18,021 scaffolds with a total of 94 Mb, in size similar to the 101 Mb Gloin1 assembly of *R. irregularis* DAOM197198 [25]. An analysis of genome completeness using the BUSCO software tool [28] showed comparable levels for both genomes (Additional file 1: Table S1). Therefore, we estimate the complete *R. clarus* MUCL46238 genome to be slightly smaller than the 150 Mb total genome size predicted for *R. irregularis* DAOM197198 [25, 45, 46].

To determine the level of conservation within the AMF symbiotic effectome*,* we compared protein sequences encoded by selected housekeeping genes and proteins involved in symbiosis from *R. irregularis* and *R. clarus* and calculated the level of identity and similarity by BLASTP (bl2seq) (Align Sequences Protein BLAST, BLASTP) (Additional file 1: Table S3). The identity of selected proteins varied between 84 % for ammonium transporter 1 to 99 % for the conserved elongation factor 1-alpha, whereas the similarity varied between 91 and 99 %, respectively. The high levels of protein identity and similarity confirm the close relationship between these two species of the same genus, which is in agreement with current knowledge [10].

Subsequently, we searched within the *R. clarus* genome for homologs of the *R. irregularis* proteins matching at least one effector criterion. Approx. 95 % (208) of *R. irregularis* effector candidates showed BLASTP (bl2seq) detectable hits within the *R. clarus* genome draft (Fig. 1). The surprisingly high number of putative effector protein homologs present in both *Rhizophagus* species clearly speaks in favour of a conserved role during the infection process and demonstrates the potential of a comparative approach.

Although the *R. clarus* draft genome is not complete and therefore incomplete ORF information in the homolog sets were to be expected, analysis of the overlapping effectome could still reveal potential roles in symbiosis. For 30 % of the effector candidates predicted in *R. irregularis* we found only truncated homolog sequences in *R. clarus*. This can only be partly explained by genome incompleteness, because the two genome assemblies were of comparable quality (Additional file 1: Table S1). A further 25 % of candidates in *R. clarus* had to be omitted because the inaccurate annotation of the *R. irregularis* proteome resulted in selection of false positive candidates in the effectome, as properly annotated *R. clarus* homologs of these candidates did not fulfil effectome criteria. Additionally, even though *R. clarus* effector protein homologs share sequence similarity with that of *R. irregularis*, they do not necessarily have a similar topology. Therefore, after applying the same selection criteria used on the *R. irregularis* secretome, where proteins predicted to hold SP, NLS, RCP but lacking TMD and ER retention signal were kept, the *R. clarus* candidate number was reduced by 13 %. The final number of full-length sequence candidates fulfilling effector selection criteria present in *R. clarus* as well as in *R. irregularis* genome was 64, which is approx. 32 % of all candidate homologs present in both species (Fig. 1). The protein similarity between the homologs was 40–98 %.

Within the 64 *R. clarus* effector candidates we identified only five proteins that fulfilled two of the effector criteria and none that matched all three of them (Additional file 1: Table S4). Furthermore, the similarity between all effector candidates from both *Rhizophagus* species was ranged from 51 % for NLS/SCR_9486 (Additional file 1: Table S4) to 97 % for NLS_7749 as determined by the BLASTP (bl2seq) algorithm (Table 1). From these, 18 conserved effector candidates show more than 90 % similarity between *R. clarus* and *R. irregularis*, which falls within the same range as the similarity between housekeeping genes (Additional file 1: Table S3). More than 44 % (8) of the conserved candidates belong to the NLS group (Fig. 1, Table 1). In addition, the NLS group shows the highest conservation level (47 % of candidates are conserved) in comparison to the SCR and RCP groups (20 % and 23 % conserved, respectively). These findings suggest that the effectors translocated to the plant nucleus with possible effect on the transcriptional regulation in plant cells might be essential for AM symbiosis. The highly conserved effector candidates identified here between different AMF species are probably playing a fundamental role during fungus-plant interaction also explaining the degree of unspecific interaction with all potential hosts. In contrast, effector candidates showing high diversification may have minor host specific functions.

**Table 1:** The *R. clarus* effector candidates with predicted function

Predicted effector	Length (aa)	Similarity %	Homology to (e-value/homology lenght)	Conserved domains	Cellular role	Putative function
Interference with signal transduction						
NLS_26232	592	91	Salt-inducible protein kinase (NP_001105276.1) *Zea mays* 4.43e^−26^/259)	Protein kinase-like, PKc (7.5e^−34^)	Cell envelope	Interference with signal transduction
NLS_30765	542	92	No significant hits	F-box-like domain (2.8e^−07^)	Cell envelope	Interference with signal transduction
NLS_334409	614	95	Nitrogen permease regulator 3-like protein isoform X1 (XP_004596640.1) Ochotona princeps (9e^−47^/595)	Nitrogen Permease regulator of amino acid transport activity 3 (9e^−88^)	Cell envelope	Interference with signal transduction
NLS_320155	274	89	PLC-like phosphodiesterase (EJF62321.1) *Dichomitus squalens (*2e^−41^/256)	Glycerophosphodiester phosphodiesterase, GDPD (3.0e^−53^)	Cell envelope	Interference with signal transduction
RCP_340423	175	88	Phospholipase d1 (EFX04791.1) *Grosmannia clavigera* (7e^−07^/98)		Cell envelope	Interference with signal transduction
SCR_348911	85	53	Peptidoglycan-binding LysM (YP_001663206.1) *Thermoanaerobacter sp. X514* (2e^−07^/53)	LysM domain (3.5e^−12^)	Cell envelope	Interference with signal transduction
NLS_7749	827	97	Trehalose phosphate synthase (EKD17284.1) *Marssonina brunnea* (0.0e/790)	Bifunctional trehalose-6-phosphate synthase/HAD hydrolase subfamily IIB domain (0.0e)	Cell envelope	Interference with signal transduction
SCR_343180	150	85	No significant hits	ML (MD-2-related lipid-recognition) (2e^−07^)	Cell envelope	Interference with signal transduction
Cell wall modification						
NLS/RCP_349824	610	83	Galactose oxidase (CCO36399.1) Rhizoctonia solani (1.8e^−70^/549)	Glyoxal oxidase N-terminus domain (1e^−29^)	Cell envelope	Plant cell wall modification
RCP_230436	569	89	class I alpha-mannosidase protein (EOD46381.1) *Neofusicoccum parvum* (1.8e^−134^/523)	Glycosyl hydrolase family 47 (0.0e)	Cell envelope	Fungal cell wall modification
RCP_343321	399	90	Chitin deacetylase (XP_003194854.1) *Cryptococcus gattii* (3e^−64^/289)	Chitin deacetylase domain (1.4e^−33^)	Cell envelope	Fungal cell wall modification
RCP_84949	326	70	Bifunctional xylanase/deacetylase (CCO26450) *Rhizoctonia solani* (4e^−55^/246)	Catalytic NodB homology domain of the carbohydrate esterase 4 superfamily (1.8e^−87^)	Cell envelope	Fungal cell wall modification
				Hevein/Chitin binding domain (3.8e^−10^)		
Transcription regulation						
NLS_98735	263	95	Hypothetical protein (EIE87396.1) *Rhizopus delemar* (8e^−13^/105)	PWWP domain (1.5e^−18^)	Transport and binding	Transcription regulation
Unknown function						
NLS_32583	730	91	DENN domain containing protein (EJY85661.1) *Oxytricha trifallax* (1e^−49^/498)	ATPase family associated with various cellular activities (AAA) (1.5e^−05^)	Amino acid biosynthesis	
NLS_343100	654	96	P-loop containing nucleoside triphosphate hydrolase protein (EIW63580.1) *Trametes versicolor* (5e^−34^/394)		Cell envelope	
SCR_339199	125	74	Carbohydrate-binding module family 19 protein (XP_001874952.1) *Laccaria bicolor* (4e^−13^/88)		Transport and binding	
SCR_94594	137	79	Proline-rich protein (XP_001875220.1) *Laccaria bicolor* (8e^−23^/ 78)		Cell envelope	
RCP_349288	473	61	Carbohydrate-binding module family 19 protein (XP_001874952.1) *Laccaria bicolor* (9e^−17^/81)		Cell envelope	
RCP_335225	347	84	Hypothetical protein (EPZ34955) *Rozella allomycis* (2e^−72^/315)		Cell envelope	

### Potential functional features of *R. clarus* effector candidates

To deduct putative functions of *R. clarus* and *R. irregularis* predicted effector candidates the BLAST2GO software with automated annotation was used [39]. The obtained information was used to predict putative cellular functions of *Rhizophagus* effector candidates (Table 1).

**Fig. 2:**
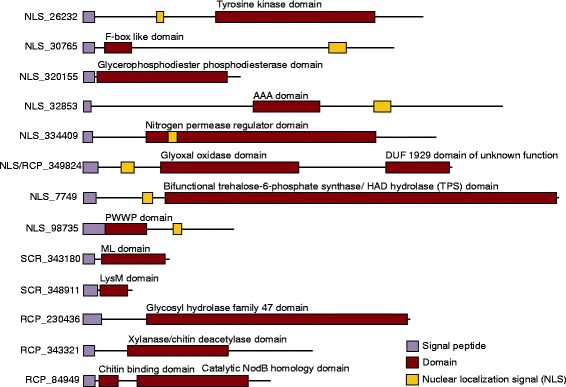
Illustration of the various functional modules of *R. clarus* effector candidates

Only 14 of *R. clarus* effector candidates (22 %) showed sequence similarity (BLASTP, cut-off e-value > 10^−5^) to documented proteins with defined or predicted function, and 13 (20 %) contain known domains or motifs (Fig. 2). Predicted domain and homology hits with an e-value higher than 10^−5^ were ignored [47]. Current research showed that a number of fungal and oomycete effectors despite being under high diversifying or positive selective pressure still carry recognizable Pfam domains, which might be helpful in prediction of their biological function [48, 49]. Thus, for 13 (20 %) of the *R. clarus* effector candidates the putative function could be determined, hence their potential role in AM symbiosis was defined and subdivided into three main functional groups: signalling, cell wall modification, and transcriptional regulation. However, the majority of *R. clarus* effector candidates are novel sequences showing no significant homology to known proteins in other organisms or sequence motifs (Additional file 1: Table S4), which is in agreement with previous studies highlighting the evolutionary diverse nature of fungal effector groups [36]. Based on computational approaches, we established effector candidate subcategories and speculated on putative functions after careful confirmation of presence of active sites and completeness of the predicted domains.

#### Secreted proteins likely to target plant signalling

Secreted effectors can alter plant defence by interfering with signal transduction [50]. Within the *R. clarus* effectome we detected eight effector candidates that might affect plant signalling.

Within the *R. clarus* effectome, candidate SCR_348911 shows homology to the *Thermoanaerobacter sp.* peptidoglycan-binding LysM protein and contains a LysM domain (PF01476). Plant receptor kinases containing a LysM (lysine motif) domain are able to detect chitin-like components or chitin fragments [51]. Chitin, an N-acetyl-glucosamine (GlcNAc) polymer, is a main component of the fungal cell wall [52]. Chitin recognition activates signalling pathways, which lead to plant immune defence [53]. Due to this, it is suggested that many plant pathogens secrete effectors that contain LysM domains in order to inhibit the release of chitin fragments from fungal cell walls or to bind these fragments, thus, preventing their recognition by plant receptors. For instance, the rice blast fungus *Magnaporthe oryzae* secretes the LysM domain containing effector, Slp1, which binds chitin oligosaccharides. This suppresses plant defence response induced through the recognition of chitin fragments by the rice receptor CEBiP (chitin elicitor binding protein) [51, 52]. Candidate SCR_348911 might play a similar role in AM symbiosis.

The second effector candidate NLS_30765 contains a F-box-like domain (PF12937). Proteins containing this domain are components of the E3 ubiquitin ligases called SCF complexes (Skp, Cullin, F-box containing complex) which control many intracellular regulatory processes by catalysing the ubiquitination of proteins that targets proteins for degradation [54]. Intense analysis showed that plants defence response pathways might be under control of an SCF complex [54, 55]. Many pathogenic effectors use a strategy of ubiquitination in order to regulate plant immunity. The AvrPiz-t effector of the *M. oryzae* binds to and destabilizes the rice RING E3 ubiquitin ligase APIP6 to suppress chitin-induced plant immunity [56].

The third effector candidate NLS_334409 shows homology to the *Ochotona princeps* nitrogen permease regulator 3-like protein (NPR3) isoform X1 and contains a NPR3 domain (PF03666) typical of this protein family. NPR3 forms a heterodimer complex with NPR2 (nitrogen permease regulator 2) protein, which mediates an amino acid starvation signal to TORC1 (target of rapamycin kinase) [57]. TORC1, an essential serine/threonine protein kinase, incorporates signals from diverse pathways in the cell. The homology of candidate NLS_334409 to NPR3 protein suggests its interference with signal transduction most likely during amino acid deficiency conditions.

A similar role in interfering with plant signalling pathways might have the fourth candidate NLS_26232 in which a protein kinases catalytic domain, PKc (PF07714) was detected. PKc domains can transfer a phosphate group from ATP to serine/threonine or tyrosine residues on protein substrates. PKs often function as components of signal transduction pathways in which one kinase activates another kinase. This serial action transfers a signal within a cell, which results in cellular responses [58].

Two additional candidates might also influence the plant signalling cascades by being involved in hydrolytic processes. The candidate NLS_320155 shows homology to the *Dichomitus squalens* PLC-like (phospholipase C-like) phosphodiesterase and has a glycerophosphodiester phosphodiesterase domain, GDPD (PF03009). PLC, by hydrolysing phosphodiester bonds, integrates signalling cascades critical for a variety of cellular and physiological functions [59]. The candidate RCP_340423 shows homology to the *Grosmannia clavigera* phospholipase d1, which catalyse the cleavage of phospholipids into fatty acids and other lipophilic substances, like phosphatidic acid, which is an established intracellular signalling lipid [60, 61].

Candidate number seven, NLS_7749, has homology to the glycosyltransferase family 20 protein from *Colletotrichum higginsianum* and encompasses a complex bifunctional domain, which includes a trehalose-6-phosphate synthase (TPS) domain (PF02358) and a HAD-superfamily hydrolase domain, subfamily IIB (TIGR0148). TPS catalyses the synthesis of α,α-1,1-trehalose-6-phosphate from glucose-6-phosphate using a UDP-glucose donor and is a key enzyme in the trehalose synthesis pathway [62]. Furthermore, trehalose was reported to have a role as a stress protectant in many organisms across kingdoms [63]. Remarkably, in pathogens trehalose is associated with several mechanisms involved in cell wall integrity, regulation of metabolism, and successful infection [64]. Absence of a trehalose metabolic apparatus is correlated with lower pathogen virulence. However, yet the underlying mechanism remains unravelled. For instance, in *M. oryzae* Tps1 regulates transcriptional effectors linked to virulence factors via NADPH-binding during appressorium-mediated rice infection [65]. Consequently, the putative effector NLS_7749 might take part in the colonization step of AMF symbiosis by being involved in trehalose synthesis.

The last candidate, SCR_343180, contains a ML (MD-2-related lipid-recognition, PF02221) domain, which is involved in specific lipid recognition. It is predicted that ML domain containing proteins through interacting with specific lipids can mediate diverse biological functions, like lipid metabolism, host response to pathogen components like lipopolysaccharides, and other cellular functions [66]. This suggests that the SCR_343180 candidate, by interacting with lipids, can interfere with the plant lipid signalling; however the direct mechanism of this effect needs to be clarified.

### Genes encoding putative secreted enzymes engaged in cell wall modification

We identified four *R. clarus* effector candidates encoding putative secreted enzymes involved in cell wall modification. They were divided in two subgroups depending whether their putative biological functions concern plant or fungal cell wall.

#### Enzymes involved in degradation of the plant cell wall

Most of the necrotrophic fungi degrade plant tissues by producing a broad set of carbohydrate-active enzymes specifically focused on plant polysaccharide degradation [67]. Transcriptomic profiling of *R. irregularis* DAOM197198 predicted 139 genes encoding carbohydrate-active enzymes [15]. This amount is relatively low in comparison to obligate biotrophic pathogens and ectomycorrhizal symbionts [68]. Also, only one putative enzyme involved in plant cell wall modification was found in the *R. clarus* effector repertoire. This suggests that during AM symbiosis the plant cell wall is not completely degraded, but instead a localized corruption of its structure might occur to make it more flexible, possibly for fungus penetration into the plant cell.

The single effector of this subgroup, candidate NLS/RCP_349824 shows homology to the *Rhizoctonia solani* galactose oxidase and contains a glyoxal oxidase domain (PF07250). Glyoxal oxidase has a copper active site remarkably similar to that found in another fungal metalloenzyme, galactose oxidase [69]. Glyoxal oxidase is a copper metalloenzyme that catalyses the oxidation of aldehydes to carboxylic acids and is an essential component of the extracellular lignin degradation pathways [70]. Although lignin is present only in small amounts in the plant cell wall [71], it has a strong impact on the rigidity of its structure. Lignin degradation might result in a more flexible cell wall necessary for fungal penetration.

#### Enzymes involved in modification of the fungal cell wall

The effectome also contains three putative enzymes involved in chitin degradation. This suggests constant remodelling of the fungal cell wall, likely an adaptation to substantial differences in environmental conditions in the soil and inside of plant tissues. Similarly, in *Cryptococcus neoformans* it was shown that chitosan, the product of chitin deacetylation, helps to sustain cell integrity by maintaining normal capsule width during vegetative growth [72].

The first effector candidate in *R. clarus*, RCP_230436, contains a glycosyl hydrolase family 47 domain (PF01532) and shows homology to the *Neofusicoccu parvum* class I alpha-mannosidase enzyme. This enzyme is involved in the cleavage of the terminal mannose from Asn-linked oligosaccharides, which takes part at the early step of hydrolysis of N-glycan such as chitin [73].

Two other *R. clarus* effector candidates might function in fungal cell wall remodelling by chitin deacetylation [74]. Candidate RCP_343321 shows homology to xylase/chitin deacetylase enzyme from *Cryptococcus gatti* and contains a chitin deacetylase domain (PF01522). The other candidate, RCP_84949, shows a significant homology to *Rhizoctonia solani* bifunctional xylanase/deacetylase. The presence of hevein/chitin binding domain (PF03067) and a catalytic NodB homology domain of the carbohydrate esterase 4 superfamily (PF01074), which typically occur in chitin deacetylases [74], suggest a chitin degradation function of both candidates.

However, candidates with putative chitin deacetylase function might also have another purpose. It was shown that chitin deacetylase plays a significant role in protecting pathogenic fungal hyphae from being lysed by plant secreted chitinases, specifically in the cases of the wheat stem rust fungus *Puccinia graminis* f. sp. *tritici* and the broad bean rust fungus *Uromyces fabae* [74]. Furthermore, it was suggested that exposed fungal chitin polymers are partially de-*N*-acetylated by chitin deacetylases during the infection of the host, this with purpose to avoid plant antimicrobial hydrolases by affinity modulation [75].

#### Genes encoding proteins with putative transcriptional regulatory function

During the pathogenic or symbiotic infection thousands of genes are up- or down-regulated in the plant as revealed by several transcriptome analyses. Expression of genes encoding enzymes involved in protein degradation, cell wall modification, and secondary metabolite biosynthesis is induced in order to activate plant immune response [62]. However, pathogens are able to secrete the so-called transcription activation-like effectors, which target DNA and are involved in transcriptional regulation of a set of plant host genes, for instance to prevent defence response [76].

We found only one putative candidate within the *R. clarus* effectome that might regulate plant gene expression during establishment of the AM symbiosis. This is the nuclear localized (data not shown) candidate, NLS_98735, which consists of a PWWP domain (PF00855), named after a conserved Pro-Trp-Trp-Pro motif. PWWP domain-containing proteins bind to chromatin and control its packing resulting in transcription and replication regulation. Moreover, PWWP proteins are also involved in DNA damage response. While DNA damage occurs, they regulate chromatin condensation and ability of DNA repair factors, like 53BPI and CtIP to bind DNA [77]. Similarly, candidate NLS_98735 might be involved in transcription regulation by chromatin remodelling or condensation.

#### Effector candidates with unknown function

Six of the *R. clarus* effector candidates show homologies to known proteins or contain identified domains or motifs. However, the similarity does not allow for prediction of a specific function. One of the candidates, NLS_32583, has an AAA (PF00004, ATPases Associated with diverse cellular Activities) domain and shows significant homology to a protozoa protein containing a DENN (Differentially Expressed in Neoplastic vs. Normal cells) domain (1e^−49^). However, the homology is mostly based on the AAA domain region as a DENN domain was not detected. The AAA superfamily is a large and functionally diverse superfamily composed of members that are essential for a variety of cellular functions, like protein unfolding and degradation, DNA recombination, replication and repair, and signal transduction [78].

Candidates SCR_94594 and RCP_335225 show homologies to putative conserved fungal proteins with unknown function. Both of them lack of any functional domain; therefore, their putative function cannot be predicted. Candidate NLS_343100 shows homology to the *Trametes versicolor* P-loop protein containing a nucleoside triphosphate hydrolase domain (P-loop NTPase). Nevertheless, this homology is partial and does not include the characteristics for this family of P-loop NTPase domain proteins. In addition, two other candidates, SCR_339199 and RCP_349288, show homology to the *Laccari bicolor* carbohydrate binding module family 19 protein (CBM19). Proteins belonging to this family contain a module of 60–70 residues (PF03427) with a chitin binding function [79]. However, once more, this module is absent in both candidates; hence, despite the homology the putative function cannot be predicted.

## Conclusions

Symbiotic interaction between AMF and plant host requires a well-adjusted dialog between the two partners in order to function properly. We hypothesise that one of the key components of this communication is the fungal effectome.

In this study, we used a bioinformatic pipeline to predict effector candidates from two AMF belonging to the *Rhizophagus* genus, *R. irregularis* and *R. clarus*. Our *in silico* pipeline revealed a list of several candidate effector proteins that create a valuable source of information to elucidate the mechanism of plant infection and colonization by AM fungi.

Our *in silico* characterization of the AMF effectome focused on *R. clarus* candidates. The majority of the *R. clarus* effectors are novel proteins with no significant homology to known sequences or motifs in other organisms. This finding is in agreement to previous reports enhancing the evolution diverse nature of fungal and oomycete effectors [34]. However, for a small number of candidates putative functions during plant-fungus symbiosis might be predicted.

The set of putative effector candidates encoded by the *R. clarus* effectome appears to be suited for a fungus that wants to establish a symbiotic rather than a pathogenic relation with the host. The presence of only one candidate encoding a putative plant cell wall degrading enzyme (PCWDE) in the *R. clarus* effectome is in agreement with earlier reports, in which it was shown that several obligate biotrophic pathogens and ectomycorrhizal symbionts have a decreased repertoire of PCWDEs compared to saprophytes [25, 67, 80, 81]. In comparison to pathogenic fungi, AMF seem not to degrade the plant cells but rather grow between them or inside of them while trying to avoid recognition by the plant defence. However, localized plant cell wall modelling probably does occur to weaken and make it more flexible during fungal penetration and passage from cell to cell. The lack of the typical pathogenic effectors, such as extracellular lipases, proteases, nucleases and phytases, additionally confirms the non-pathogenic status of AMF [82].

The relationship between symbiotic fungi and plants requires a major rearrangement of the plant signalling pathways for which fungi have evolved a range of secreted proteins. For instance, fungal effectors control auxin signalling within plant cells to alter plant root architecture and favour colonization [50]. Thus, the presence of candidates in the *R. clarus* effectome that might target plant signalling in order to allow fungal entry was expected. Reprogramming of the plant defence could be used to avoid recognition. Alternatively, effectors might affect transport processes across the plant cell membrane. This was previously shown for symbiotic fungi which encode effectors to control the transfer of glucose into the apoplastic space of the root where it is easily accessible for fungus [50].

A remarkable aspect of our work is presence of a large portion of the effectors that are not species-specific, but conserved between different *Rhizophagus* species. This group of highly conserved effector proteins may play a fundamental role during fungus-plant interaction and has first priority for future functional characterization.

### Availability of supporting data

The sequences of the predicted effector candidates have been deposited in GenBank under accession numbers: KU305736 - KU305799 (as presented in Additional file 1: Table S4). Other supporting data are included in an Additional file 1.

## Additional file

Additional file 1: Figure S1. Gene Ontology category analyses by ProtFun 2.2 server [40] of *R. irregularis* DAOM197198 secretome and effectome. **Table S1.** *R. clarus* draft genome assembly statistics in comparison to published *R. irregularis* genome assemblies. **Table S2.** The *R. irregularis* putative effectome. **Table S3.** Identity and similarity levels between *R. irregularis* and *R. clarus* based on sequences of selected proteins (Align Sequences Protein BLAST, BLASTP). **Table S4.** The *R. clarus* effector candidates. (DOCX 152 kb)

## Abbreviations

AAA: ATPases associated with diverse cellular activities
AM: Arbuscular mycorrhizal
AMF: Arbuscular mycorrhizal fungi
CEBiP: Chitin elicitor binding protein
DENN: differentially expressed in neoplastic vs. normal cells
ER: endoplasmic reticulum
ETI: effector-triggered immunity
GDPD: glycerophosphodiester phosphodiesterase domain
GlcNAc: N-acetyl-glucosamine
LysM: lysine motif
*M. oryzae*: *Magnaporthe oryzae*
Mio: million
ML: MD-2-related lipid-recognition
Mya: million years ago
NB-LRR: nucleotide-binding site leucine-rich repeat
NLS: nuclear localization signal
NPR2: nitrogen permease regulator 2
NPR3: nitrogen permease regulator 3-like protein
PCWDE: plant cell wall degrading enzyme
PKc: protein kinases catalytic domain
PLC-like: phospholipase C-like
P-loop NTPase: P-loop protein with a nucleoside triphosphate hydrolase domain
PWWP: Pro-Trp-Trp-Pro
QC: quality control
*R. clarus*: *Rhizophagus clarus*
*R. irregularis*: *Rhizophagus irregularis*
RCP: repeats containing proteins
SCR: small cysteine-rich
SP: signal peptide
SP7: secreted effector protein 7
TMD: transmembrane domain
TORC1: target of rapamycin kinase
TPS: trehalose-6-phosphate synthase
