# Supplementary material for: The effector candidate repertoire of the arbuscular mycorrhizal fungus Rhizophagus clarus
Source: BMC Genomics. 2016 Feb 9;17:101. doi: 10.1186/s12864-016-2422-y (PMC4746824; doi:10.1186/s12864-016-2422-y)
Supplement: Additional file 1: Figure S1. — Gene Ontology category analyses by ProtFun 2.2 server [40] of R. irregularis DAOM197198 secretome and effectome. Table S1. R. clarus draft genome assembly statistics in comparison to published R. irregularis genome assemblies. Table S2. The R. irregularis putative effectome. Table S3. Identity and similarity levels between R. irregularis and R. clarus based on sequences of selected proteins (Align Sequences Protein BLAST, BLASTP). Table S4. The R. clarus effector candidates. (DOCX 152 kb) [file 12864_2016_2422_MOESM1_ESM.docx]

## Supporting Information

Article title: ***The effector candidate repertoire of the arbuscular mycorrhizal fungus* Rhizophagus clarus**

Authors: Kinga A. Sędzielewska and Andreas Brachmann

The following Supporting Information is available for this article:

**Fig. S1** Gene Ontology category analyses by ProtFun 2.2 server [42] of *R. irregularis* DAOM197198 secretome and effectome

**Table S1** *R. clarus* draft genome assembly statistics in comparison to published *R. irregularis* genome assemblies

| **Parameters** | ***R. clarus* MUCL46238 draft genome assembly** | ***R. irregularis* DAOM197198 genome assembly Gloin1 from Tisserant et al. [25]** | ***R. irregularis* DAOM197198w genome assembly from Lin et al. [46]** |
| --- | --- | --- | --- |
| Number of reads | 71,796,102 | 30,067,076 | 206,988,880 |
| Raw sequence data | 8,232 Mb | 3,781 Mb | 21,500 Mb |
| Number of scaffolds | 18,021 | 28,371 | 30,638 |
| Assembly size | 94 Mb | 101 Mb | 141 Mb |
| N50 | 8.79 kb | 4.19 kb | 16.36 kb |
| **BUSCO genome benchmarks*** |  |  |  |
| Complete single-copy BUSCOs | 82% (1,184/1,438) | 84% (1,219/1,438) | 76% (1,096/1,438) |
| Complete duplicated BUSCOs | 8.1% (117/1,438) | 8.1% (117/1,438) | 8.5% (123/1,438) |
| Fragmented BUSCOs | 11% (159/1,438) | 8.6% (125/1,438) | 11% (165/1,438) |
| Missing BUSCOs | 6.6% (95/1,438) | 6.5% (94/1,438) | 12% (177/1,438) |

* Analysis of BUSCO genome benchmarks was performed according to Simão et al. [28].

**Table S2** The *R. irregularis* putative effectome

|  | **Predicted effector** | **Length (aa)** | **Homology to  (e value/homology length)** | | **Conserved domain** | **Cellular role** | **Putative function** |
| --- | --- | --- | --- | --- | --- | --- | --- |
| **NLS** | | | | | | | |
| 1 | jgi\|Gloin1\|10232 | 258 | CenpB-DNA-bind-domain-containing protein (EIM19112.1) *Wallemia sebi CBS 633.66* (8e^-14^/123) | | Homeodomain-like (8.96e^-06^) | Translation |  |
| 2 | jgi\|Gloin1\|1128 | 134 | No significant hits | |  | Transport and binding | Signal transducer |
| 3 | jgi\|Gloin1\|130297 | 784 | No significant hits | |  | Cell envelope |  |
| 4 | jgi\|Gloin1\|14050 | 123 | No significant hits | |  | Translation | Growth factor |
| 5 | jgi\|Gloin1\|147264 | 89 | No significant hits | | Protein kinase-like (2e^-09^) | Energy metabolism | Stress response |
| 6 | jgi\|Gloin1\|154898 | 717 | Transmembrane protein (XP_001828627.2)  *Coprinopsis cinerea okayama*7#130 (5e^-32^/139) | | C2 domain (Calcium/lipid-binding domain) (4.12e^-35^) | Cell envelope | Immune response |
| 7 | jgi\|Gloin1\|15886 | 275 | Hypothetical protein RO3G_04810 (EIE80105.1) *Rhizopus delemar* RA 99-880 (5.5e^-06^/275) | | zf-CCHC Zinc knuckle (3.3e^-07^) | Translation | Growth factor |
| 8 | jgi\|Gloin1\|167440 | 501 | NADH-cytochrome b5 reductase 1, partial (ELR46439.1) *Bos grunniens mutus* (3.97e^-15^/241) | | Cytochrome b5-like Heme/Steroid binding domain (4.32e^-13^) | Central intermediary metabolism |  |
|  |  |  |  |  | Oxidoreductase FAD-binding domain (7.2e^-14^) |  |  |
| 9 | jgi\|Gloin1\|18384 | 214 | Hypothetical protein (EJF64529.1)  *Dichomitus squalens* (4.13e^-15^/128) | |  | Cell envelope | Growth factor |
| 10 | jgi\|Gloin1\|19095 | 220 | No significant hits | |  | Cell envelope | Stress response |
| 11 | jgi\|Gloin1\|19392 | 469 | Hypothetical protein SELMODRAFT_404776 (XP_002962747.1)  *Selaginella moellendorffii* (1.03e^-22^/363) | |  | Cell envelope | Stress response |
| 12 | jgi\|Gloin1\|19919 | 170 | No significant hits | | Myb-like DNA-binding domain (3.1e^-09^) | Replication and transcription | Transcription |
| 13 | jgi\|Gloin1\|21321 | 88 | No significant hits | | Ring finger domain (1e^-04^) | Fatty acid metabolism | Growth factor |
| 14 | jgi\|Gloin1\|217541 | 479 | No significant hits | |  | Cell envelope |  |
| 15 | jgi\|Gloin1\|227 | 373 | No significant hits | |  | Cell envelope | Immune response |
| 16 | jgi\|Gloin1\|23087 | 88 | No significant hits | |  | Cell envelope | Immune response |
| 17 | jgi\|Gloin1\|23326 | 134 | No significant hits | |  |  |  |
| 18 | jgi\|Gloin1\|23630 | 188 | No significant hits | |  |  |  |
| 19 | jgi\|Gloin1\|23994 | 229 | Thiamine biosynthetic bifunctional enzyme (ENH78363.1) *Colletotrichum orbiculare* (3.08e^-15^/229) | | LysM domain (8,37e^-08^) |  |  |
| 20 | jgi\|Gloin1\|26232 | 592 | Hypothetical protein PRUPE_ppa020252mg (EMJ27490.1) *Prunus persica* (9.16e^-24^/311) | | Protein kinase domain (1.3e^-23^) | Cell envelope |  |
| 21 | jgi\|Gloin1\|26361 | 116 | No significant hits | |  |  |  |
| 22 | jgi\|Gloin1\|27056 | 601 | Putative dna repair and recombination protein rad54 (EOD49553.1) *Neofusicoccum parvum* (0.0e / 600) | | SNF2 family N-terminal domain (1.5e^-75^) | Central intermediary metabolism |  |
|  |  |  |  |  | Helicase conserved C-terminal domain (1.5e^-27^) |  |  |
| 23 | jgi\|Gloin1\|27162 | 107 | No significant hits | |  | Transport and binding |  |
| 24 | jgi\|Gloin1\|29498 | 632 | No significant hits | |  | Cell envelope | Immune response |
| 25 | jgi\|Gloin1\|30438 | 107 | No significant hits | |  |  |  |
| 26 | jgi\|Gloin1\|30594 | 267 | Hypothetical protein (WP_001405030.1)  *Escherichia coli* (3.07e^-135^/266) | |  |  |  |
| 27 | jgi\|Gloin1\|30765 | 543 | No significant hits | | F-box-like (7e^-08^) | Cell envelope |  |
| 28 | jgi\|Gloin1\|320155 | 278 | Glycerophosphodiesterase (AFR95900.1) *Cryptococcus neoformans var. grubii* H99 (5.09e^-39^/237) | | Glycerophosphoryl diester phosphodiester (2.49e^-55^) | Cell envelope | Hormone |
| 29 | jgi\|Gloin1\|324984 | 204 | Contains similarity to *Arabidopsis thaliana* far-red impaired response protein (AAF88018.1)  *Arabidopsis thaliana* (5.14e^-04^/145) | |  |  |  |
| 30 | jgi\|Gloin1\|32583 | 464 | Hypothetical protein BRAFLDRAFT_106560 (XP_002599417.1) *Branchiostoma floridae* (3.4e^-97^/464) | | P-loop containing nucleoside triphosphate hydrolases superfamily (6.64e^-12^) | Amino acid biosynthesis |  |
| 31 | jgi\|Gloin1\|330 | 484 | No significant hits | |  | Central intermediary metabolism |  |
| 32 | jgi\|Gloin1\|334409 | 611 | Npr2/3 complex subunit Npr3 (predicted) (NP_596792.1)  *Schizosaccharomyces pombe* (9.2e^-49^/611) | | Nitrogen Permease regulator of amino acid transport activity 3 (6.31e^-91^) | Cell envelope |  |
| 33 | jgi\|Gloin1\|335765 | 246 | Hypothetical protein RO3G_04998 (EIE80293.1) *Rhizopus delemar* RA 99-880 (2.11e^-15^/126) | | CUE domain (3.1e^-10^) |  |  |
| 34 | jgi\|Gloin1\|33649 | 635 | Serine/threonine protein kinase (EGD78136.1) *Salpingoeca* sp. ATCC 50818 (1.39e^-61^/635) | | P-loop containing nucleoside triphosphate hydrolases superfamily (1.69e^-15^) | Energy metabolism |  |
| 35 | jgi\|Gloin1\|337567 | 385 | Hypothetical protein RO3G_09249  *Rhizopus delemar* (4.8e^-21^/216) | |  | Cell envelope |  |
| 36 | jgi\|Gloin1\|338930 | 79 | No significant hits | |  |  |  |
| 37 | jgi\|Gloin1\|339987 | 545 | Endoplasmic oxidoreductin 1 precursor (EJT52204.1) *Trichosporon asahii var. Asahii* (2e^-122^/522) | | ERO1 Endoplasmic Reticulum Oxidoreductin 1 (1.0e^-132^) | Cell envelope |  |
| 38 | jgi\|Gloin1\|340836 | 123 | Centromere protein S (EMT65185.1)  *Fusarium oxysporum* f. sp. cubense (4.1e^-16^/79) | |  |  |  |
| 39 | jgi\|Gloin1\|342866 | 362 | Putative u11/U12 small nuclear ribonucleoprotein  35 kDa protein-like (XP_001375255.1)  *Monodelphis domestica* (2.99e^-62^/169) | | RNA recognition motif (1.7e^-24^) | Replication and transcription | Immune response |
| 40 | jgi\|Gloin1\|343100 | 431 | P-loop containing nucleoside triphosphate hydrolase protein (EJD00138.1)  *Fomitiporia mediterranea* MF3/22 (6.88e^-31^/390) | |  | Cell envelope |  |
| 41 | jgi\|Gloin1\|34944 | 147 | No significant hits | |  | Energy metabolism | Growth factor |
| 42 | jgi\|Gloin1\|36410 | 51 | No significant hits | |  |  |  |
| 43 | jgi\|Gloin1\|4050 | 265 | No significant hits | |  |  |  |
| 44 | jgi\|Gloin1\|62 | 135 | No significant hits | |  |  |  |
| 45 | jgi\|Gloin1\|6251 | 576 | Conserved hypothetical protein (XP_002380620.1) *Aspergillus flavus* (3e^-04^/57) | |  | Cell envelope | Stress response |
| 46 | jgi\|Gloin1\|7749 | 845 | Glycosyltransferase family 20 (CCF39482.1) *Colletotrichum higginsianum* (0.0e/845) | | Glycosyltransferase family 20 (1.1e^-172^) | Cell envelope |  |
|  |  |  |  |  | Trehalose-phosphatase (1.2e^-64^) |  |  |
| 47 | jgi\|Gloin1\|86791 | 224 | No significant hits | |  |  |  |
| 48 | jgi\|Gloin1\|8769 | 60 | No significant hits | |  |  |  |
| 49 | jgi\|Gloin1\|90403 | 697 | Chromosome segregation protein sudA (EON65649.1) *Coniosporium apollinis* CBS 100218 (0.0e/657) | | Chromosome segregation ATPases Smc hinge domain (1.75e^-56^) |  |  |
| 50 | jgi\|Gloin1\|90856 | 545 | Hypothetical protein RO3G_07595 (EIE82890.1) *Rhizopus delemar* RA 99-880 (2.5e^-15^/299) | |  |  |  |
| 51 | jgi\|Gloin1\|91924 | 153 | No significant hits | |  |  |  |
| 52 | jgi\|Gloin1\|9486 | 146 | No significant hits | |  | Cell envelope |  |
| 53 | jgi\|Gloin1\|96244 | 262 | No significant hits | |  |  |  |
| 54 | jgi\|Gloin1\|96688 | 291 | No significant hits | |  |  |  |
| 55 | jgi\|Gloin1\|97100 | 241 | CRN-like CRN11 (AAY43405.1)  *Phytophthora infestans* (2.75e^-07^/122) | |  |  |  |
| 56 | jgi\|Gloin1\|98735 | 250 | Hypothetical protein RO3G_12107 (EIE87396.1) *Rhizopus delemar* RA 99-880 (2.6e^-13^/105) | | PWWP domain (1.8e^-19^) | Transport and binding | Immune response |
| **SCR** | | | | | | | |
| 57 | jgi\|Gloin1\|10822 | 89 | No significant hits | |  |  |  |
| 58 | jgi\|Gloin1\|11377 | 72 | No significant hits | |  | Transport and binding | Hormone |
| 59 | jgi\|Gloin1\|11576 | 89 | Membrane protein (WP_000825670.1)  *Escherichia* sp. TW09308 (9.32e^-23^/50) | | Prokaryotic membrane lipoprotein lipid attachment site (6.5e^-04^) |  |  |
| 60 | jgi\|Gloin1\|11869 | 52 | No significant hits | |  |  |  |
| 61 | jgi\|Gloin1\|121071 | 78 | No significant hits | |  |  |  |
| 62 | jgi\|Gloin1\|12350 | 126 | No significant hits | |  |  |  |
| 63 | jgi\|Gloin1\|137350 | 77 | No significant hits | |  |  |  |
| 64 | jgi\|Gloin1\|147264 | 89 | No significant hits | | Protein kinase-like (2e^-09^) |  |  |
| 65 | jgi\|Gloin1\|14763 | 118 | No significant hits | |  | Cell envelope | Hormone |
| 66 | jgi\|Gloin1\|15964 | 149 | No significant hits | |  |  |  |
| 67 | jgi\|Gloin1\|16629 | 64 | No significant hits | |  |  |  |
| 68 | jgi\|Gloin1\|168 | 95 | Hypothetical protein (XP_001439256.1)  *Paramecium tetraurelia* (3.81e^-05^/57) | | Plexin repeat (1.9e^-04^) | Cell envelope | Immune response |
| 69 | jgi\|Gloin1\|17381 | 60 | No significant hits | |  |  |  |
| 70 | jgi\|Gloin1\|18179 | 80 | No significant hits | |  | Cell envelope | Signal transducer |
| 71 | jgi\|Gloin1\|182238 | 103 | Hypothetical protein (XP_001903763.1)  *Podospora anserina* (2.8e^-19^/101) | |  | Cell envelope | Signal transducer |
| 72 | jgi\|Gloin1\|19130 | 59 | No significant hits | |  |  |  |
| 73 | jgi\|Gloin1\|194116 | 142 | No significant hits | |  | Cell envelope | Growth factor |
| 74 | jgi\|Gloin1\|19646 | 93 | No significant hits | |  |  |  |
| 75 | jgi\|Gloin1\|19895 | 125 | No significant hits | |  |  |  |
| 76 | jgi\|Gloin1\|206588 | 136 | No significant hits | |  |  |  |
| 77 | jgi\|Gloin1\|20753 | 57 | No significant hits | |  |  |  |
| 78 | jgi\|Gloin1\|20809 | 50 | Histidine kinase-, DNA gyrase b-, and HSP90-like ATPase domain-containing protein (ELU37460.1) *Rhizoctonia solani* AG-1 IA (6.1e^-07^/42) | |  |  |  |
| 79 | jgi\|Gloin1\|20931 | 117 | No significant hits | |  |  |  |
| 80 | jgi\|Gloin1\|21321 | 88 | No significant hits | | Zinc finger, C3HC4 type (1e^-04^) |  |  |
| 81 | jgi\|Gloin1\|21353 | 148 | No significant hits | |  |  |  |
| 82 | jgi\|Gloin1\|22936 | 80 | No significant hits | |  | Cell envelope | Hormone |
| 83 | jgi\|Gloin1\|23231 | 84 | No significant hits | |  |  |  |
| 84 | jgi\|Gloin1\|248224 | 123 | No significant hits | |  |  |  |
| 85 | jgi\|Gloin1\|25081 | 132 | No significant hits | |  | Cell envelope | Hormone |
| 86 | jgi\|Gloin1\|25359 | 62 | Sugar transporter (AEK82125.1)  *Rhizophagus intraradices* (1.23e^-15^/53) | | MFS general substrate transporter (4.05e^-04^) | Cell envelope | Structural protein |
| 87 | jgi\|Gloin1\|25613 | 97 | No significant hits | |  |  |  |
| 88 | jgi\|Gloin1\|257852 | 77 | No significant hits | |  |  |  |
| 89 | jgi\|Gloin1\|26361 | 116 | No significant hits | |  |  |  |
| 90 | jgi\|Gloin1\|26992 | 124 | No significant hits | |  |  |  |
| 91 | jgi\|Gloin1\|281646 | 130 | No significant hits | |  | Cell envelope | Stress response |
| 92 | jgi\|Gloin1\|28912 | 141 | No significant hits | |  | Cell envelope | Growth factor |
| 93 | jgi\|Gloin1\|29643 | 50 | No significant hits | |  |  |  |
| 94 | jgi\|Gloin1\|29701 | 53 | No significant hits | | Cytochrome P450 (4.85e^-06^) |  |  |
| 95 | jgi\|Gloin1\|30350 | 101 | No significant hits | |  | Cell envelope | Hormone |
| 96 | jgi\|Gloin1\|30692 | 150 | No significant hits | |  |  |  |
| 97 | jgi\|Gloin1\|30919 | 141 | No significant hits | |  |  |  |
| 98 | jgi\|Gloin1\|31474 | 90 | No significant hits | |  |  |  |
| 99 | jgi\|Gloin1\|31643 | 114 | No significant hits | |  | Cell envelope | Hormone |
| 100 | jgi\|Gloin1\|31656 | 113 | No significant hits | |  | Transport and binding | Immune response |
| 101 | jgi\|Gloin1\|31657 | 120 | No significant hits | |  |  |  |
| 102 | jgi\|Gloin1\|319075 | 139 | No significant hits | |  | Energy metabolism | Hormone |
| 103 | jgi\|Gloin1\|319617 | 111 | No significant hits | |  |  |  |
| 104 | jgi\|Gloin1\|32041 | 116 | No significant hits | |  | Cell envelope | Growth factor |
| 105 | jgi\|Gloin1\|32399 | 74 | No significant hits | |  | Cell envelope | Hormone |
| 106 | jgi\|Gloin1\|326976 | 116 | No significant hits | |  |  |  |
| 107 | jgi\|Gloin1\|32918 | 116 | No significant hits | |  | Cell envelope | Hormone |
| 108 | jgi\|Gloin1\|336365 | 133 | No significant hits | |  | Cell envelope | Hormone |
| 109 | jgi\|Gloin1\|336696 | 103 | No significant hits | |  |  |  |
| 110 | jgi\|Gloin1\|337029 | 82 | No significant hits | |  |  |  |
| 111 | jgi\|Gloin1\|337358 | 61 | No significant hits | |  |  |  |
| 112 | jgi\|Gloin1\|337637 | 54 | No significant hits | |  |  |  |
| 113 | jgi\|Gloin1\|337906 | 108 | Hypothetical protein AGABI1DRAFT_99591 (EKM79972.1)  *Agaricus bisporus var. burnettii* JB137-S8 (5.6e^-05^/74) | |  | Cell envelope | Stress response |
| 114 | jgi\|Gloin1\|338978 | 95 | No significant hits | |  |  |  |
| 115 | jgi\|Gloin1\|339199 | 133 | Carbohydrate-binding module family 19 protein (XP_001874952.1)  *Laccaria bicolor* S238N-H82 (4.37e^-16^/91) | |  | Transport and binding | Immune response |
| 116 | jgi\|Gloin1\|339961 | 58 | No significant hits | |  |  |  |
| 117 | jgi\|Gloin1\|341325 | 62 | No significant hits | |  | Cell envelope | Hormone |
| 118 | jgi\|Gloin1\|341327 | 63 | No significant hits | |  |  |  |
| 119 | jgi\|Gloin1\|341967 | 107 | No significant hits | |  | Cell envelope | Hormone |
| 120 | jgi\|Gloin1\|342813 | 143 | No significant hits | |  | Cell envelope | Hormone |
| 121 | jgi\|Gloin1\|343180 | 150 | No significant hits | | ML (MD-2-related lipid-recognition) domain (2.2e^-08^) | Cell envelope | Immune response |
| 122 | jgi\|Gloin1\|343733 | 67 | No significant hits | |  | Transport and binding | Hormone |
| 123 | jgi\|Gloin1\|343772 | 103 | No significant hits | |  |  |  |
| 124 | jgi\|Gloin1\|343985 | 53 | No significant hits | |  | Transport and binding | Hormone |
| 125 | jgi\|Gloin1\|345636 | 61 | No significant hits | |  | Transport and binding | Hormone |
| 126 | jgi\|Gloin1\|34619 | 108 | No significant hits | |  |  |  |
| 127 | jgi\|Gloin1\|346314 | 104 | No significant hits | |  |  |  |
| 128 | jgi\|Gloin1\|346946 | 119 | No significant hits | |  | Cell envelope | Stress response |
| 129 | jgi\|Gloin1\|347068 | 57 | No significant hits | |  | Energy metabolism | Growth factor |
| 130 | jgi\|Gloin1\|347085 | 95 | No significant hits | |  | Cell envelope | Receptor |
| 131 | jgi\|Gloin1\|347147 | 53 | No significant hits | |  |  |  |
| 132 | jgi\|Gloin1\|347272 | 82 | No significant hits | |  | Transport and binding | Hormone |
| 133 | jgi\|Gloin1\|347635 | 61 | No significant hits | |  |  |  |
| 134 | jgi\|Gloin1\|348043 | 149 | No significant hits | | ML (MD-2-related lipid-recognition) domain (2.6e^-09^) | Cell envelope | Immune response |
| 135 | jgi\|Gloin1\|348911 | 82 | Peptidoglycan-binding protein (WP_003867981.1) *Thermoanaerobacter ethanolicus* (4.5e^-08^/55) | | LysM domain (5e^-11^) | Cell envelope | Hormone |
| 136 | jgi\|Gloin1\|349137 | 54 | No significant hits | |  |  |  |
| 137 | jgi\|Gloin1\|349993 | 95 | No significant hits | |  |  |  |
| 138 | jgi\|Gloin1\|35048 | 64 | No significant hits | |  |  |  |
| 139 | jgi\|Gloin1\|35179 | 139 | No significant hits | |  |  |  |
| 140 | jgi\|Gloin1\|35548 | 88 | No significant hits | |  |  |  |
| 141 | jgi\|Gloin1\|3763 | 105 | Hypothetical protein (WP_001348068.1)  *Escherichia coli* (1.3e^-65^/104) | |  |  |  |
| 142 | jgi\|Gloin1\|4615 | 94 | No significant hits | |  |  |  |
| 143 | jgi\|Gloin1\|4655 | 112 | No significant hits | |  | Energy metabolism | Stress response |
| 144 | jgi\|Gloin1\|4656 | 113 | No significant hits | |  |  |  |
| 145 | jgi\|Gloin1\|54675 | 51 | Predicted protein (XP_001876473.1)  *Laccaria bicolor* S238N-H82 (2.04e^-05^/51) | | hAT family dimerisation domain (3.3e^-05^) | Energy metabolism | Hormone |
| 146 | jgi\|Gloin1\|67794 | 100 | Hypothetical protein (XP_001903763.1)  *Podospora anserina* (1.6e^-14^/99) | |  | Cell envelope | Signal transducer |
| 147 | jgi\|Gloin1\|7716 | 72 | No significant hits | |  |  |  |
| 148 | jgi\|Gloin1\|83509 | 135 | No significant hits | |  |  |  |
| 149 | jgi\|Gloin1\|84793 | 113 | No significant hits | |  |  |  |
| 150 | jgi\|Gloin1\|84949 | 112 | Chitin binding protein (XP_001933394.1)  *Pyrenophora tritici-repentis* Pt-1C-BFP (4.58e^-09^/112) | |  | Cell envelope | Immune response |
| 151 | jgi\|Gloin1\|86848 | 102 | No significant hits | |  | Transport and binding | Signal transducer |
| 152 | jgi\|Gloin1\|8744 | 72 | No significant hits | |  | Transport and binding | Stress response |
| 153 | jgi\|Gloin1\|8819 | 102 | No significant hits | |  |  |  |
| 154 | jgi\|Gloin1\|89182 | 127 | No significant hits | |  | Transport and binding | Stress response |
| 155 | jgi\|Gloin1\|91334 | 132 | No significant hits | |  |  |  |
| 156 | jgi\|Gloin1\|94594 | 121 | Proline-rich protein (XP_001875220.1)  *Laccaria bicolor* (1.2e^-12^/92) | |  | Cell envelope | Stress response |
| 157 | jgi\|Gloin1\|9486 | 146 | No significant hits | |  |  |  |
| 158 | jgi\|Gloin1\|9670 | 57 | No significant hits | |  |  |  |
| 159 | jgi\|Gloin1\|98724 | 132 | No significant hits | |  |  |  |
| 160 | jgi\|Gloin1\|99199 | 110 | Hypothetical protein (XP_002837605.1)  *Tuber melanosporum* (1.57e^-05^/57) | |  | Cell envelope | Hormone |
| **RCP** | | | | | | | |
| 161 | jgi\|Gloin1\|10685 | 90 | | No significant hits |  | Cell envelope | Immune response |
| 162 | jgi\|Gloin1\|10686 | 282 | | No significant hits |  |  |  |
| 163 | jgi\|Gloin1\|11122 | 120 | | No significant hits |  |  |  |
| 164 | jgi\|Gloin1\|1128 | 134 | | No significant hits |  |  |  |
| 165 | jgi\|Gloin1\|11353 | 163 | | No significant hits |  |  |  |
| 166 | jgi\|Gloin1\|12665 | 362 | | No significant hits |  | Cell envelope | Immune response |
| 167 | jgi\|Gloin1\|12736 | 230 | | No significant hits |  | Cell envelope | Immune response |
| 168 | jgi\|Gloin1\|151076 | 104 | | No significant hits |  |  |  |
| 169 | jgi\|Gloin1\|154898 | 717 | | Transmembrane protein (XP_001828627.2) *Coprinopsis cinerea okayama*7#130 (3.7e^-31^/139) |  |  |  |
| 170 | jgi\|Gloin1\|16976 | 204 | | No significant hits |  | Transport and binding | Growth factor |
| 171 | jgi\|Gloin1\|17708 | 212 | | No significant hits | Kelch motif (7.7e^-11^) |  |  |
| 172 | jgi\|Gloin1\|18384 | 214 | | Hypothetical protein (EJF64529.1)  *Dichomitus squalens* LYAD-421 SS1 (4.1e^-15^/128) |  |  |  |
| 173 | jgi\|Gloin1\|1942 | 920 | | No significant hits | Protein kinase domain (2.7e^-06^) |  |  |
| 174 | jgi\|Gloin1\|19684 | 206 | | No significant hits |  | Cell envelope | Growth factor |
| 175 | jgi\|Gloin1\|19919 | 170 | | No significant hits | Myb-like DNA-binding domain (3.1e^-09^) |  |  |
| 176 | jgi\|Gloin1\|206588 | 136 | | No significant hits |  | Cell envelope | Hormone |
| 177 | jgi\|Gloin1\|21353 | 148 | | No significant hits |  |  |  |
| 178 | jgi\|Gloin1\|217882 | 110 | | No significant hits |  |  |  |
| 179 | jgi\|Gloin1\|223029 | 157 | | No significant hits |  | Cell envelope | Growth factor |
| 180 | jgi\|Gloin1\|227 | 373 | | No significant hits |  |  |  |
| 181 | jgi\|Gloin1\|22936 | 80 | | No significant hits |  |  |  |
| 182 | jgi\|Gloin1\|230436 | 625 | | Class I alpha-mannosidase (XP_002484908.1) *Talaromyces stipitatus* ATCC 10500 (4.07e^-130^/512) | Gycosyl hydrolase family 47 (0.0e) | Cell envelope |  |
| 183 | jgi\|Gloin1\|23087 | 88 | | No significant hits |  |  |  |
| 184 | jgi\|Gloin1\|23558 | 75 | | No significant hits |  |  |  |
| 185 | jgi\|Gloin1\|25081 | 132 | | No significant hits |  |  |  |
| 186 | jgi\|Gloin1\|26449 | 80 | | No significant hits |  |  |  |
| 187 | jgi\|Gloin1\|26454 | 161 | | No significant hits |  |  |  |
| 188 | jgi\|Gloin1\|29498 | 632 | | No significant hits |  |  |  |
| 189 | jgi\|Gloin1\|30919 | 141 | | No significant hits |  | Cell envelope | Hormone |
| 190 | jgi\|Gloin1\|318823 | 403 | | No significant hits |  |  |  |
| 191 | jgi\|Gloin1\|322019 | 123 | | No significant hits |  |  |  |
| 192 | jgi\|Gloin1\|3238 | 450 | | No significant hits |  | Cell envelope | Immune response |
| 193 | jgi\|Gloin1\|324984 | 204 | | Contains simlarity to *Arabidopsis thaliana* far-red impaired response protein (AAF88018.1)  *Arabidopsis thaliana* (5.14e^-04^/145) |  |  |  |
| 194 | jgi\|Gloin1\|3337 | 288 | | No significant hits |  |  |  |
| 195 | jgi\|Gloin1\|335225 | 387 | | Endoglucanase (EGE03508.1)  *Trichophyton equinum* CBS 127.97 (9.68e^-23^/236) |  | Cell envelope | Growth factor |
| 196 | jgi\|Gloin1\|339987 | 545 | | Endoplasmic oxidoreductin 1 precursor (EJT52204.1) *Trichosporon asahii var. asahii*  (2e^-122^/522) | Endoplasmic Reticulum Oxidoreductin 1 (ERO1) (1.2e^-131^) |  |  |
| 197 | jgi\|Gloin1\|340423 | 169 | | Phospholipase d1 (EFX04791.1)  *Grosmannia clavigera* kw1407 (9.48e^-05^/50) |  | Cell envelope | Stress response |
| 198 | jgi\|Gloin1\|343321 | 372 | | Hypothetical protein RO3G_12908 (EIE88197.1) *Rhizopus delemar* RA 99-880 (1.83e^-64^/268) | Xylanase/chitin deacetylase (5.61e^-34^) | Cell envelope | Stress response |
| 199 | jgi\|Gloin1\|345355 | 383 | | Hypothetical protein RO3G_12908 (EIE88197.1) *Rhizopus delemar* RA 99-880 (7.05e^-70^/263) | Xylanase/chitin deacetylase (1.98e^-35^) | Central intermediary metabolism | Immune response |
| 200 | jgi\|Gloin1\|34674 | 63 | | No significant hits |  | Cell envelope | Hormone |
| 201 | jgi\|Gloin1\|347485 | 201 | | No significant hits |  |  |  |
| 202 | jgi\|Gloin1\|348533 | 76 | | No significant hits |  | Transport and binding | Hormone |
| 203 | jgi\|Gloin1\|348888 | 134 | | Hypothetical protein (WP_010181975.1)  *Aquimarina agarilytica* (8.4e^-16^/74) |  |  |  |
| 204 | jgi\|Gloin1\|349288 | 430 | | Carbohydrate-binding module family 19 protein (XP_001874952.1)  *Laccaria bicolor* S238N-H82 (1.99e^-17^/82) |  | Cell envelope | Signal transducer |
| 205 | jgi\|Gloin1\|349646 | 84 | | No significant hits |  |  |  |
| 206 | jgi\|Gloin1\|349745 | 204 | | No significant hits |  | Cell envelope | Immune response |
| 207 | jgi\|Gloin1\|349824 | 608 | | Copper radical oxidase (XP_001833162.2) *Coprinopsis cinerea okayama*7#130 (7.04e^-81^/496) | Galactose (/glyoxal) oxidase (1.31e^-29^) | Cell envelope | Stress response |
| 208 | jgi\|Gloin1\|350186 | 302 | | Hypothetical protein Rta_26950 (YP_004619815.1) *Ramlibacter tataouinensis* TTB310 (2.7^-09^/181) | DUF3455 Protein of unknown function  (6.6e^-17^) | Cell envelope | Immune response |
| 209 | jgi\|Gloin1\|35179 | 139 | | No significant hits |  |  |  |
| 210 | jgi\|Gloin1\|35406 | 68 | | No significant hits |  |  |  |
| 211 | jgi\|Gloin1\|4050 | 265 | | No significant hits |  | Energy metabolism | Immune response |
| 212 | jgi\|Gloin1\|425 | 116 | | No significant hits |  |  |  |
| 213 | jgi\|Gloin1\|7062 | 220 | | No significant hits |  |  |  |
| 214 | jgi\|Gloin1\|71582 | 579 | | Hypothetical protein RO3G_00712 (EIE76008.1) *Rhizopus delemar* RA 99-880 (7.5e^-114^/438) | TPR repeat, SEL1 subfamily (7.4e^-42^) |  |  |
| 215 | jgi\|Gloin1\|84831 | 222 | | No significant hits |  | Cell envelope | Stress response |
| 216 | jgi\|Gloin1\|86935 | 115 | | Hypothetical protein BcDW1_10875 (EMR80495.1) *Botryotinia fuckeliana* BcDW1 (1.7e^-06^/90) |  | Cell envelope | Transcription regulation |
| 217 | jgi\|Gloin1\|87860 | 433 | | No significant hits |  |  |  |
| 218 | jgi\|Gloin1\|90856 | 545 | | Hypothetical protein RO3G_07595 (EIE82890.1) *Rhizopus delema*r RA 99-880 (2.5e^-15^/299) |  |  |  |
| 219 | jgi\|Gloin1\|95830 | 151 | | Blue (type 1) copper domain protein (WP_009761927.1)  *Halobacterium* sp. DL1 (5.33e^-06^/48) | Cupredoxin-like domain (4.87e^-07^) |  |  |
| 220 | jgi\|Gloin1\|96025 | 275 | | No significant hits |  |  |  |

**Table S3** Identity and similarity levels between *R. irregularis* and *R. clarus* based on sequences of selected proteins (Align Sequences Protein BLAST, BLASTP)

| **Housekeeping genes** | **Length (aa) *R. irregularis*** | **Length (aa) *R. clarus*** | **Identity (%)** | **Similarity (%)** |
| --- | --- | --- | --- | --- |
| RNA polymerase II large subunit | 529 | 531 | 99 | 99 |
| Elongation factor 1-alpha | 344 | 341 | 99 | 99 |
| Ste12-like transcription factor | 530 | 529 | 98 | 99 |
| V-type H+ ATPase | 799 | 798 | 94 | 97 |
| ATP synthase | 272 | 253 | 95 | 98 |
| **Genes involved in symbiosis** | | | | |
| Phosphate transporter | 418 | 335 | 86 | 93 |
| Glutamine synthase | 355 | 355 | 95 | 98 |
| Ammonium transporter 1 | 479 | 492 | 84 | 91 |
| Ammonium transporter 2 | 470 | 472 | 89 | 95 |
| Ammonium transporter 3 | 454 | 452 | 92 | 96 |

**Table S4** The *R. clarus* effector candidates (BLAST2GO hit length)

| **Predicted effector** | | | | **GenBank number** | | **Length (aa)** | **Similarity %** | **Homology to  (e value/homology length)** | **Query coverage %** | **Conserved domains** | **Cellular role** | **Putative function** | | |
| --- | --- | --- | --- | --- | --- | --- | --- | --- | --- | --- | --- | --- | --- | --- |
|  | **NLS** | | | | | | | | | | | | | |
| NLS_130297 | | | | | KU305738 | 945 | 86 | No significant hits |  |  | Purines and pyrimidines |  | | |
| NLS/RCP_18384 | | | | | KU305736 | 319 | 77 | Expressed protein (XP_003030832.1) *Schizophyllum commune* (3e^-19^/149) | 82 |  | Cell envelope |  | | |
| NLS_26232 | | | | | KU305739 | 592 | 91 | Salt-inducible protein kinase (NP_001105276.1) *Zea mays* (4.43e^-26^/259) | 80 | Protein kinase-like, PKc (7.5e^-34^) | Cell envelope | Interference with signal transduction | | |
| NLS_29498 | | | | | KU305740 | 618 | 62 | No significant hits |  |  | Cell envelope |  | | |
| NLS_30765 | | | | | KU305741 | 542 | 92 | No significant hits |  | F-box-like domain (2.8e^-07^) | Cell envelope | Interference with signal transduction | | |
| NLS_320155 | | | | | KU305742 | 274 | 89 | PLC-like phosphodiesterase (EJF62321.1)  *Dichomitus squalens* (2e^-41^/237) | 79 | Glycerophosphodiester phosphodiesterase, GDPD (3.0e^-53^) | Cell envelope | Interference with signal transduction | | |
| NLS_32583 | | | | | KU305743 | 730 | 91 | DENN domain containing protein (EJY85661.1)  *Oxytricha trifallax* (1e^-49^/498) | 95 | ATPase family associated with various cellular activities (AAA) (1.5e^-05^) | Amino acid biosynthesis |  | | |
| NLS_330 | | | | | KU305744 | 497 | 85 | No significant hits |  |  | Central intermediary metabolism |  | | |
| NLS_334409 | | | | | KU305745 | 614 | 95 | Nitrogen permease regulator 3-like protein isoform X1 (XP_004596640.1)  *Ochotona princeps* (9e^-47^/595) | 48 | Nitrogen permease regulator of amino acid transport activity 3 (9e^-88^) | Cell envelope | Interference with signal transduction | | |
| NLS_337567 | | | | | KU305746 | 373 | 79 | Hypothetical protein (RO3G_09249) *Rhizopus delemar* (3e^-21/^213) | 75 |  | Cell envelope |  | | |
| NLS_343100 | | | | | KU305747 | 654 | 96 | P-loop containing nucleoside triphosphate hydrolase protein (XP007339181.1) *Auricularia subglabra* (8e^-37^/225) | 47 |  | Cell envelope |  | | |
| NLS_34944 | | | | | KU305748 | 234 | 93 | No significant hits |  |  | Energy metabolism |  | | |
| NLS/RCP_349824 | | | | | KU305752 | 610 | 83 | Galactose oxidase (CCO36399.1) *Rhizoctonia solani* (1.8e^-70^/549) | 64 | Glyoxal oxidase N-terminus domain (1e^-29^) | Cell envelope | Cell wall modification | | |
| NLS_6251 | | | | | KU305749 | 550 | 85 | No significant hits |  |  | Cell envelope |  | | |
| NLS_7749 | | | | | KU305750 | 827 | 97 | Trehalose phosphate synthase (EKD17284.1) *Marssonina brunnea* (0.0e/790) | 59 | Bifunctional trehalose-6-phosphate synthase/HAD hydrolase subfamily IIB domain (0.0e) | Cell envelope | Metabolic function | | |
| NLS/SCR_9486 | | | | | KU305737 | 226 | 51 | No significant hits |  |  | Cell envelope |  | | |
| NLS_98735 | | | | | KU305751 | 263 | 95 | Hypothetical protein (EIE87396.1)  *Rhizopus delemar* (8e^-13^/105) | 7 | PWWP domain (1.5e^-18^) | Transport and binding | Transcription/ translation regulation | | |
|  | |  | **SCR** | | | | | | | | | |  |  |
| SCR_11377 | | | | | KU305766 | 72 | 84 | No significant hits |  |  | Transport and binding |  | | |
| SCR_14763 | | | | | KU305767 | 117 | 56 | Hypothetical protein (XP_002837605.1) *Tuber melanosporum* (2e^-04^/53) | 50 |  | Cell envelope |  | | |
| SCR_18179 | | | | | KU305768 | 81 | 68 | No significant hits |  |  | Cell envelope |  | | |
| SCR_182238 | | | | | KU305769 | 103 | 81 | Hypothetical protein (XP_001903763.1) *Podospora anserina* (18e^-16^/101) | 98 |  | Cell envelope |  | | |
| SCR/RCP_206588 | | | | | KU305774 | 105 | 78 | No significant hits |  |  | Cell envelope |  | | |
| SCR_22936 | | | | | KU305775 | 72 | 71 | No significant hits |  |  | Cell envelope |  | | |
| SCR_25081 | | | | | KU305776 | 114 | 71 | No significant hits |  |  | Cell envelope |  | | |
| SCR_30350 | | | | | KU305777 | 108 | 75 | No significant hits |  |  | Cell envelope |  | | |
| SCR/RCP_30919 | | | | | KU305778 | 113 | 65 | No significant hits |  |  | Cell envelope |  | | |
| SCR_31643 | | | | | KU305779 | 118 | 87 | No significant hits |  |  | Cell envelope |  | | |
| SCR_31656 | | | | | KU305780 | 123 | 91 | No significant hits |  |  | Transport and binding |  | | |
| SCR_319075 | | | | | KU305781 | 146 | 82 | No significant hits |  |  | Energy metabolism |  | | |
| SCR_32041 | | | | | KU305782 | 94 | 78 | No significant hits |  |  | Cell envelope |  | | |
| SCR_32918 | | | | | KU305783 | 114 | 79 | No significant hits |  |  | Cell envelope |  | | |
| SCR_336365 | | | | | KU305784 | 122 | 89 | No significant hits |  |  | Cell envelope |  | | |
| SCR_337906 | | | | | KU305764 | 117 | 78 | Hypothetical protein (XP_002837605.1) *Tuber melanosporum* (2.8e^-04^/52) | 45 |  | Cell envelope |  | | |
| SCR_339199 | | | | | KU305770 | 125 | 74 | Carbohydrate-binding module family 19 protein (XP_001874952.1) *Laccaria bicolor* (4e^-13^/88) | 62 |  | Transport and binding |  | | |
| SCR_341325 | | | | | KU305785 | 63 | 92 | No significant hits |  |  | Cell envelope |  | | |
| SCR_341967 | | | | | KU305786 | 114 | 82 | No significant hits |  |  | Cell envelope |  | | |
| SCR_342813 | | | | | KU305787 | 143 | 82 | No significant hits |  |  | Cell envelope |  | | |
| SCR_343180 | | | | | KU305771 | 150 | 85 | No significant hits |  | ML (MD-2-related lipid-recognition) (2e^-07^) | Cell envelope | Metabolic function | | |
| SCR_343733 | | | | | KU305798 | 59 | 87 | No significant hits |  |  | Transport and binding |  | | |
| SCR_343985 | | | | | KU305797 | 52 | 71 | No significant hits |  |  | Transport and binding |  | | |
| SCR_345636 | | | | | KU305788 | 66 | 91 | No significant hits |  |  | Transport and binding |  | | |
| SCR_347085 | | | | | KU305789 | 107 | 70 | No significant hits |  |  | Cell envelope |  | | |
| SCR_347272 | | | | | KU305790 | 126 | 88 | No significant hits |  |  | Transport and binding |  | | |
| SCR_348911 | | | | | KU305772 | 85 | 53 | Peptidoglycan-binding LysM (YP_001663206.1)  *Thermoanaerobacter sp. X514*  (2e^-07^/53) | 58 | LysM domain (3.5e^-12^) | Cell envelope | Interference with signal transduction | | |
| SCR_4655 | | | | | KU305791 | 123 | 91 | No significant hits |  |  | Energy metabolism |  | | |
| SCR_67794 | | | | | KU305792 | 102 | 89 | Hypothetical protein (XP_001903763.1) *Podospora anserina* (8e^-17^/100) | 99 |  | Cell envelope |  | | |
| SCR_86848 | | | | | KU305793 | 102 | 95 | No significant hits |  |  | Transport and binding |  | | |
| SCR_8744 | | | | | KU305794 | 71 | 93 | No significant hits |  |  | Transport and binding |  | | |
| SCR_89182 | | | | | KU305795 | 128 | 92 | No significant hits |  |  | Transport and binding |  | | |
| SCR_94594 | | | | | KU305773 | 137 | 79 | Proline-rich protein (XP_001875220.1)  *Laccaria bicolor* (8e^-23^/ 78) | 56 |  | Cell envelope |  | | |
| SCR_99199 | | | | | KU305796 | 117 | 73 | Hypothetical protein (XP_002837605.1) *Tuber melanosporum* (2e^-04^/52) | 45 |  | Cell envelope |  | | |
|  | |  | **RCP** | | | | | | | | | | |  |
| RCP_10685 | | | | KU305799 | | 147 | 66 | No significant hits |  |  | Cell envelope |  | | |
| RCP_16976 | | | | KU305758 | | 207 | 94 | No significant hits |  |  | Transport and binding |  | | |
| RCP_19684 | | | | KU305759 | | 195 | 86 | No significant hits |  |  | Cell envelope |  | | |
| RCP_230436 | | | | KU305753 | | 569 | 89 | class I alpha-mannosidase protein (EOD46381.1)  *Neofusicoccum parvum* (1.8e^-134^/523) | 47 | Glycosyl hydrolase family 47 (0.0e) | Cell envelope | Cell wall modification | | |
| RCP_335225 | | | | KU305754 | | 347 | 84 | Hypothetical protein (EPZ34955) *Rozella allomycis* (2e^-72^/315) | 67 |  | Cell envelope |  | | |
| RCP_340423 | | | | KU305755 | | 175 | 88 | Phospholipase d1 (EFX04791.1) *Grosmannia clavigera* (7e^-07^/98) | 33 |  | Cell envelope | Interference with signal transduction | | |
| RCP_343321 | | | | KU305756 | | 399 | 90 | Chitin deacetylase (XP_003194854.1) *Cryptococcus gattii* (3e^-64^/289) | 63 | Chitin deacetylase domain (1.4e^-33^) | Cell envelope | Cell wall modification | | |
| RCP_348533 | | | | KU305760 | | 65 | 75 | No significant hits |  |  | Transport and binding |  | | |
| RCP_349288 | | | | KU305757 | | 473 | 61 | Carbohydrate-binding module family 19 protein (XP_001874952.1)  *Laccaria bicolor* (9e^-17^/81) | 34 |  | Cell envelope |  | | |
| RCP_349745 | | | | KU305761 | | 207 | 90 | No significant hits |  |  | Cell envelope |  | | |
| RCP_4050 | | | | KU305763 | | 229 | 86 | No significant hits |  |  | Energy metabolism |  | | |
| RCP_84831 | | | | KU305762 | | 222 | 83 | No significant hits |  |  | Cell envelope |  | | |
| RCP_84949 | | | | KU305765 | | 326 | 70 | Bifunctional xylanase/deacetylase (CCO26450) *Rhizoctonia solani* (4e^-55^/246) | 50 | Catalytic NodB homology domain of the carbohydrate esterase 4 superfamily  (1.8e^-87^) | Cell envelope | Cell wall modification | | |
|  |  |  |  |  | |  |  |  |  | Hevein/Chitin binding domain  (3.8e^-10^) |  |  |  |  |
